# Correction: Signatures of somatic mutations and gene expression from p16^INK4A^ positive head and neck squamous cell carcinomas (HNSCC)

**DOI:** 10.1371/journal.pone.0308819

**Published:** 2024-08-08

**Authors:** Nabil F. Saba, Ashok R. Dinasarapu, Kelly R. Magliocca, Bhakti Dwivedi, Sandra Seby, Zhaohui S. Qin, Mihir Patel, Christopher C. Griffith, Xu Wang, Mark El-Deiry, Conor Ernst Steuer, Jeanne Kowalski, Dong Moon Shin, Michael E. Zwick, Zhuo Georgia Chen

Fig 6 is incorrect. The authors have provided a corrected version here.

**Fig 6:**
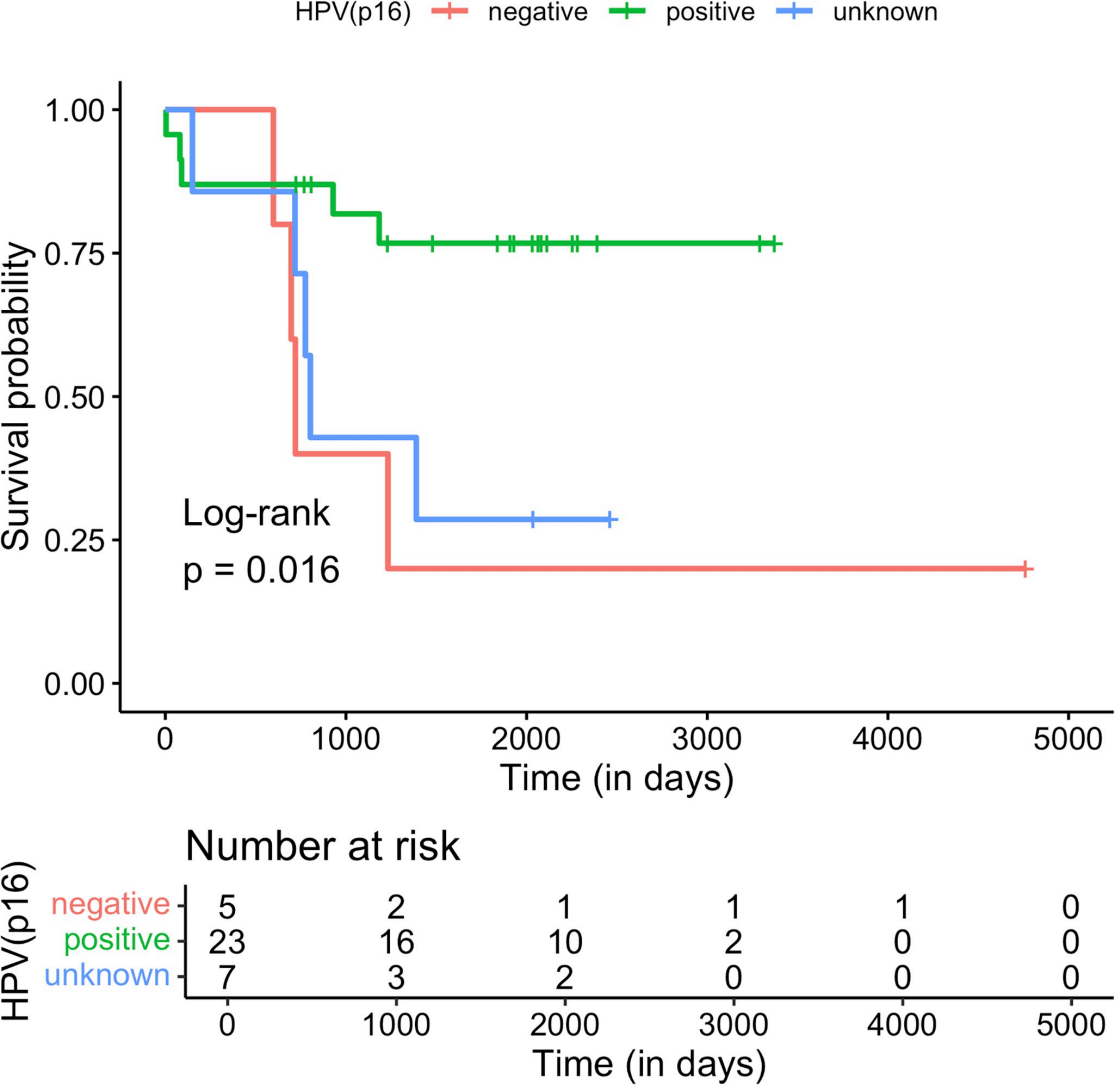
Kaplan-Meier curves of the molecular subtypes of HNSCC cohort based on p16 status.
