# Supplementary material for: Signatures of somatic mutations and gene expression from p16INK4A positive head and neck squamous cell carcinomas (HNSCC)
Source: PLoS One. 2020 Sep 28;15(9):e0238497. doi: 10.1371/journal.pone.0238497 (PMC7521680; doi:10.1371/journal.pone.0238497)
Supplement: S1 Table — (DOCX) [file pone.0238497.s001.docx]

**Table S1**

| **Sample_ID** | **p16-status** | **DNA-seq** | **RNA-seq** | **Age** | **Race** | **Sex** | **Anatomy** | **Smoking** | **Radiation (Yes/No)** | **Chemo (Yes/No)** |
| --- | --- | --- | --- | --- | --- | --- | --- | --- | --- | --- |
| GHN-62 | Negative | Y | Y | 71 | white | male | BOT | Never | Y | N |
| GHN-48 | Negative | Y | Y | 68 | white | female | BOT | Current | Y | Y |
| GHN-57 | Negative | Y | Y | 50 | white | female | BOT | Current | Y | Y |
| GHN-25 | Negative |  | Y | 63 | white | female | Tongue | Former | Y | Y |
| GHN-76 | Negative | Y | Y | 60 | AA | male | Tonsil | Former | N | N |
| GHN-77 | Positive | Y |  | 53 | AA | male | BOT | Never | N | N |
| GHN-82 | Positive | Y | Y | 67 | white | male | BOT | Former | Unknown | Unknown |
| GHN-71 | Positive | Y | Y | 59 | white | male | BOT | Never | N | Y |
| GHN-39 | Positive | Y | Y | 51 | white | male | BOT | Cannabis | Y | Y |
| GHN-4 | Positive |  | Y | 64 | white | male | BOT | Former | Y | Y |
| GHN-43 | Positive | Y |  | 65 | white | male | BOT | Former | Y | Y |
| GHN-65 | Positive | Y |  | 63 | white | male | BOT | Former | Y | Y |
| GHN-70 | Positive | Y |  | 68 | white | male | BOT | Former | Y | Y |
| GHN-69 | Positive | Y | Y | 56 | white | male | BOT | Former | Y | Y |
| GHN-60 | Positive | Y | Y | 41 | white | male | BOT | Former | Y | Y |
| GHN-64 | Positive | Y | Y | 49 | white | male | BOT | Never | Y | Y |
| GHN-83 | Positive | Y |  | 54 | white | male | Tonsil | Never | N | N |
| GHN-85 | Positive | Y |  | 56 | white | male | Tonsil | Never | Y | N |
| GHN-84 | Positive | Y | Y | 56 | white | male | Tonsil | Never | Y | N |
| GHN-79 | Positive | Y | Y | 65 | white | male | Tonsil | Former | N | Y |
| GHN-66 | Positive | Y |  | 52 | white | male | Tonsil | Current | Y | Y |
| GHN-80 | Positive | Y |  | 54 | AA | male | Tonsil | Current | Y | Y |
| GHN-68 | Positive | Y | Y | 51 | white | male | Tonsil | Current | Y | Y |
| GHN-40 | Positive | Y | Y | 68 | white | male | Tonsil | Former | Y | Y |
| GHN-73 | Positive | Y |  | 72 | white | female | Tonsil | Never | Y | Y |
| GHN-67 | Positive | Y | Y | 54 | white | male | Tonsil | Never | Y | Y |
| GHN-63 | Positive | Y |  | 65 | white | male | Unknown | Former | Y | Y |
| GHN-75 | Positive | Y |  | 61 | asian | male | Unknown | Former | Y | Y |
| GHN-20 | Unknown |  | Y | 74 | AA | male | FOM | Former | N | N |
| GHN-53 | Unknown | Y | Y | 58 | white | male | Larynx | Current | Y | Y |
| GHN-24 | Unknown |  | Y | 29 | white | female | Tongue | Former | N | N |
| GHN-21 | Unknown |  | Y | 74 | white | male | Tongue | Former | N | N |
| GHN-30 | Unknown |  | Y | 61 | white | female | Tongue | Former | Y | Y |
| GHN-15 | Unknown |  | Y | 52 | white | male | Tongue | Former | Y | Y |
| GHN-11 | Unknown |  | Y | 64 | white | female | Tongue | Never | Y | Y |
