# Supplementary material for: Signatures of somatic mutations and gene expression from p16INK4A positive head and neck squamous cell carcinomas (HNSCC)
Source: PLoS One. 2020 Sep 28;15(9):e0238497. doi: 10.1371/journal.pone.0238497 (PMC7521680; doi:10.1371/journal.pone.0238497)
Supplement: S2 Table — (DOCX) [file pone.0238497.s002.docx]

**Table S2**

|  | **All** | **p16-negative** | **p16-positive** | **Unknown** |
| --- | --- | --- | --- | --- |
| Genome Build | hg19 | hg19 | hg19 | hg19 |
| Samples | 27 | 4 | 22 | 1 |
| nGenes | 100 | 16 | 92 | 7 |
| Frame Shift Del | 9 | 2 | 7 | 0 |
| Frame Shift Ins | 5 | 2 | 4 | 1 |
| In Frame Del | 1 | 0 | 1 | 0 |
| In Frame Ins | 6 | 0 | 4 | 0 |
| Missense Mutation | 175 | 14 | 156 | 5 |
| Nonsense Mutation | 29 | 2 | 26 | 1 |
| total | 225 | 20 | 198 | 7 |
