# Supplementary material for: Signatures of somatic mutations and gene expression from p16INK4A positive head and neck squamous cell carcinomas (HNSCC)
Source: PLoS One. 2020 Sep 28;15(9):e0238497. doi: 10.1371/journal.pone.0238497 (PMC7521680; doi:10.1371/journal.pone.0238497)
Supplement: S3 Table — (DOCX) [file pone.0238497.s003.docx]

**Table S3**

| **Sample** | **p16** | **GATK4/Mutec2** | **ANNOVAR** | **ANNOVAR.exonic** | **COSMIC v87 hg19** |
| --- | --- | --- | --- | --- | --- |
| GHN_76 | Negative | 31 | 31 | 8 | 3 |
| GHN_62 | Negative | 38 | 38 | 15 | 3 |
| GHN_57 | Negative | 38 | 38 | 11 | 2 |
| GHN_48 | Negative | 39 | 39 | 15 | 8 |
| GHN_83 | Positive | 17 | 17 | 13 | 5 |
| GHN_69 | Positive | 32 | 32 | 14 | 2 |
| GHN_84 | Positive | 33 | 33 | 13 | 5 |
| GHN_79 | Positive | 33 | 33 | 8 | 4 |
| GHN_71 | Positive | 36 | 36 | 10 | 2 |
| GHN_63 | Positive | 37 | 37 | 12 | 4 |
| GHN_64 | Positive | 37 | 37 | 18 | 7 |
| GHN_60 | Positive | 38 | 38 | 15 | 2 |
| GHN_85 | Positive | 39 | 39 | 15 | 7 |
| GHN_39 | Positive | 39 | 39 | 11 | 5 |
| GHN_68 | Positive | 39 | 39 | 14 | 6 |
| GHN_65 | Positive | 47 | 47 | 10 | 5 |
| GHN_80 | Positive | 47 | 47 | 16 | 8 |
| GHN_66 | Positive | 51 | 51 | 10 | 4 |
| GHN_70 | Positive | 59 | 59 | 26 | 7 |
| GHN_73 | Positive | 61 | 61 | 19 | 4 |
| GHN_77 | Positive | 66 | 66 | 25 | 8 |
| GHN_75 | Positive | 66 | 66 | 15 | 8 |
| GHN_40 | Positive | 67 | 67 | 28 | 11 |
| GHN_82 | Positive | 78 | 78 | 26 | 7 |
| GHN_43 | Positive | 90 | 90 | 47 | 15 |
| GHN_67 | Positive | 92 | 92 | 45 | 11 |
| GHN_53 | Unknown | 64 | 64 | 21 | 11 |
