# Supplementary material for: Signatures of somatic mutations and gene expression from p16INK4A positive head and neck squamous cell carcinomas (HNSCC)
Source: PLoS One. 2020 Sep 28;15(9):e0238497. doi: 10.1371/journal.pone.0238497 (PMC7521680; doi:10.1371/journal.pone.0238497)
Supplement: S5 Table — (DOCX) [file pone.0238497.s005.docx]

**Table S5**

| **Barcode** | **Tumor_Site** | **Final_HPV_Status** | **Smoking_Status** |
| --- | --- | --- | --- |
| TCGA-CR-6472 | Base_of_Tongue | Positive | Light/Non-Smoker |
| TCGA-CR-5243 | Tonsil | Positive | Light/Non-Smoker |
| TCGA-CR-5249 | Tonsil | Positive | Light/Non-Smoker |
| TCGA-BA-5153 | Tonsil | Positive | Light/Non-Smoker |
| TCGA-CR-7385 | Tonsil | Positive | Light/Non-Smoker |
| TCGA-BA-5559 | Tonsil | Positive | Light/Non-Smoker |
| TCGA-HD-7754 | Tonsil | Positive | Light/Non-Smoker |
| TCGA-BB-4223 | Tonsil | Positive | Light/Non-Smoker |
| TCGA-CR-6481 | Tonsil | Positive | Light/Non-Smoker |
| TCGA-CR-6470 | Tonsil | Positive | Light/Non-Smoker |
| TCGA-CR-5250 | Base_of_Tongue | Positive | Smoker |
| TCGA-BA-4077 | Base_of_Tongue | Positive | Smoker |
| TCGA-CV-6433 | Oral_Tongue | Positive | Smoker |
| TCGA-CV-5971 | Oral_Tongue | Positive | Smoker |
| TCGA-CR-5248 | Tonsil | Positive | Smoker |
| TCGA-CR-6482 | Tonsil | Positive | Smoker |
| TCGA-CR-6487 | Tonsil | Positive | Smoker |
| TCGA-CN-5374 | Tonsil | Positive | Smoker |
| TCGA-BA-5557 | Oral_Tongue | Negative | Light/Non-Smoker |
| TCGA-BA-6873 | Oral_Tongue | Negative | Light/Non-Smoker |
| TCGA-CN-4725 | Oral_Tongue | Negative | Light/Non-Smoker |
| TCGA-CN-6017 | Oral_Tongue | Negative | Light/Non-Smoker |
| TCGA-CQ-6229 | Oral_Tongue | Negative | Light/Non-Smoker |
| TCGA-CQ-7065 | Oral_Tongue | Negative | Light/Non-Smoker |
| TCGA-CR-6488 | Oral_Tongue | Negative | Light/Non-Smoker |
| TCGA-CR-7372 | Oral_Tongue | Negative | Light/Non-Smoker |
| TCGA-CR-7382 | Oral_Tongue | Negative | Light/Non-Smoker |
| TCGA-CR-7393 | Oral_Tongue | Negative | Light/Non-Smoker |
| TCGA-CV-5973 | Oral_Tongue | Negative | Light/Non-Smoker |
| TCGA-CV-6003 | Oral_Tongue | Negative | Light/Non-Smoker |
| TCGA-CV-7243 | Oral_Tongue | Negative | Light/Non-Smoker |
| TCGA-CX-7085 | Oral_Tongue | Negative | Light/Non-Smoker |
| TCGA-D6-6515 | Oral_Tongue | Negative | Light/Non-Smoker |
| TCGA-DQ-5624 | Oral_Tongue | Negative | Light/Non-Smoker |
| TCGA-CV-5439 | Base_of_Tongue | Negative | Smoker |
| TCGA-CV-6950 | Base_of_Tongue | Negative | Smoker |
| TCGA-BA-4074 | Oral_Tongue | Negative | Smoker |
| TCGA-BA-7269 | Oral_Tongue | Negative | Smoker |
| TCGA-CN-4736 | Oral_Tongue | Negative | Smoker |
| TCGA-CN-4742 | Oral_Tongue | Negative | Smoker |
| TCGA-CN-5370 | Oral_Tongue | Negative | Smoker |
| TCGA-CN-6019 | Oral_Tongue | Negative | Smoker |
| TCGA-CN-6020 | Oral_Tongue | Negative | Smoker |
| TCGA-CN-6024 | Oral_Tongue | Negative | Smoker |
| TCGA-CN-6998 | Oral_Tongue | Negative | Smoker |
| TCGA-CQ-5330 | Oral_Tongue | Negative | Smoker |
| TCGA-CQ-6221 | Oral_Tongue | Negative | Smoker |
| TCGA-CQ-6224 | Oral_Tongue | Negative | Smoker |
| TCGA-CQ-6225 | Oral_Tongue | Negative | Smoker |
| TCGA-CR-7390 | Oral_Tongue | Negative | Smoker |
| TCGA-CR-7391 | Oral_Tongue | Negative | Smoker |
| TCGA-CR-7392 | Oral_Tongue | Negative | Smoker |
| TCGA-CR-7397 | Oral_Tongue | Negative | Smoker |
| TCGA-CV-5976 | Oral_Tongue | Negative | Smoker |
| TCGA-CV-6436 | Oral_Tongue | Negative | Smoker |
| TCGA-CV-6441 | Oral_Tongue | Negative | Smoker |
| TCGA-CV-6945 | Oral_Tongue | Negative | Smoker |
| TCGA-CV-6951 | Oral_Tongue | Negative | Smoker |
| TCGA-CV-6952 | Oral_Tongue | Negative | Smoker |
| TCGA-CV-6954 | Oral_Tongue | Negative | Smoker |
| TCGA-CV-7180 | Oral_Tongue | Negative | Smoker |
| TCGA-CV-7236 | Oral_Tongue | Negative | Smoker |
| TCGA-D6-6823 | Oral_Tongue | Negative | Smoker |
| TCGA-D6-6825 | Oral_Tongue | Negative | Smoker |
| TCGA-DQ-5625 | Oral_Tongue | Negative | Smoker |
| TCGA-CR-5247 | Tonsil | Negative | Smoker |
| TCGA-CR-7383 | Tonsil | Negative | Smoker |

| **Tumor_Site** | **Final_HPV_Status** | **Smoking_Status** | **#** |
| --- | --- | --- | --- |
| Oral_Tongue | Negative | Smoker | 29 |
| Oral_Tongue | Negative | Light/Non-Smoker | 16 |
| Base_of_Tongue | Negative | Smoker | 2 |
| Tonsil | Negative | Smoker | 2 |
| Tonsil | Positive | Light/Non-Smoker | 9 |
| Tonsil | Positive | Smoker | 4 |
| Base_of_Tongue | Positive | Smoker | 2 |
| Oral_Tongue | Positive | Smoker | 2 |
| Base_of_Tongue | Positive | Light/Non-Smoker | 1 |
|  |  |  | 67 |
