# Supplementary material for: Signatures of somatic mutations and gene expression from p16INK4A positive head and neck squamous cell carcinomas (HNSCC)
Source: PLoS One. 2020 Sep 28;15(9):e0238497. doi: 10.1371/journal.pone.0238497 (PMC7521680; doi:10.1371/journal.pone.0238497)
Supplement: S6 Table — (DOCX) [file pone.0238497.s006.docx]

**Table S6**

| **Barcode** | **Tumor_Site** | **RNA_type_16** | **Final_HPV_**  **Status** | **Smoking_**  **Status** | **MassArray_HPV_**  **Status** | **MassArray_HPV_**  **Call_1** |
| --- | --- | --- | --- | --- | --- | --- |
| TCGA-CR-6472 | Base of Tongue | 7802 | Positive | Light/Non-Smoker | Positive | HPV16 |
| TCGA-CR-5243 | Tonsil | 21145 | Positive | Light/Non-Smoker | Positive | HPV16 |
| TCGA-CR-5249 | Tonsil | 16981 | Positive | Light/Non-Smoker | Positive | HPV16 |
| TCGA-BA-5153 | Tonsil | 15191 | Positive | Light/Non-Smoker | Positive | HPV16 |
| TCGA-CR-7385 | Tonsil | 14859 | Positive | Light/Non-Smoker | Positive | HPV16 |
| TCGA-BA-5559 | Tonsil | 13550 | Positive | Light/Non-Smoker | Positive | HPV16 |
| TCGA-HD-7754 | Tonsil | 11667 | Positive | Light/Non-Smoker | Positive | HPV16 |
| TCGA-BB-4223 | Tonsil | 10823 | Positive | Light/Non-Smoker | Positive | HPV16 |
| TCGA-CR-6481 | Tonsil | 9451 | Positive | Light/Non-Smoker | Positive | HPV16 |
| TCGA-CR-6470 | Tonsil | 9240 | Positive | Light/Non-Smoker | Positive | HPV16 |
| TCGA-CR-5250 | Base of Tongue | 15090 | Positive | Smoker | Positive | HPV16 |
| TCGA-BA-4077 | Base of Tongue | 11271 | Positive | Smoker | Positive | HPV16 |
| TCGA-CV-6433 | Oral Tongue | 15781 | Positive | Smoker | Positive | HPV16 |
| TCGA-CV-5971 | Oral Tongue | 1980 | Positive | Smoker | Positive | HPV16 |
| TCGA-CR-5248 | Tonsil | 9419 | Positive | Smoker | Positive | HPV16 |
| TCGA-CR-6482 | Tonsil | 8602 | Positive | Smoker | Positive | HPV16 |
| TCGA-CR-6487 | Tonsil | 7844 | Positive | Smoker | Positive | HPV16 |
| TCGA-CN-5374 | Tonsil | 7074 | Positive | Smoker | Positive | HPV16 |
