# Supplementary figures and images for: Signatures of somatic mutations and gene expression from p16INK4A positive head and neck squamous cell carcinomas (HNSCC)
Source: PLoS One. 2020 Sep 28;15(9):e0238497. doi: 10.1371/journal.pone.0238497 (PMC7521680; doi:10.1371/journal.pone.0238497)

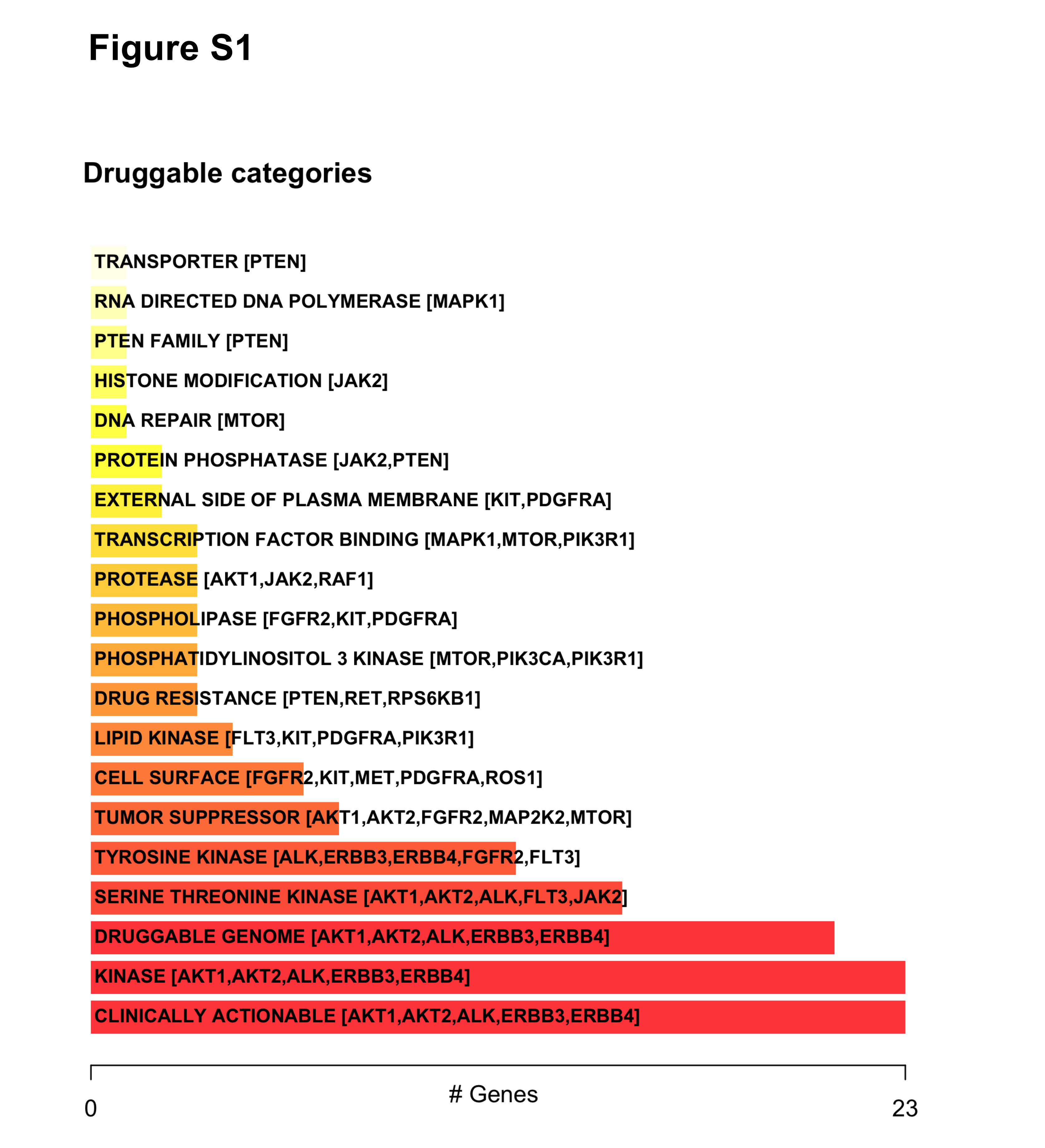

Supplement: S1 Fig — (TIF) [file pone.0238497.s007.tif]

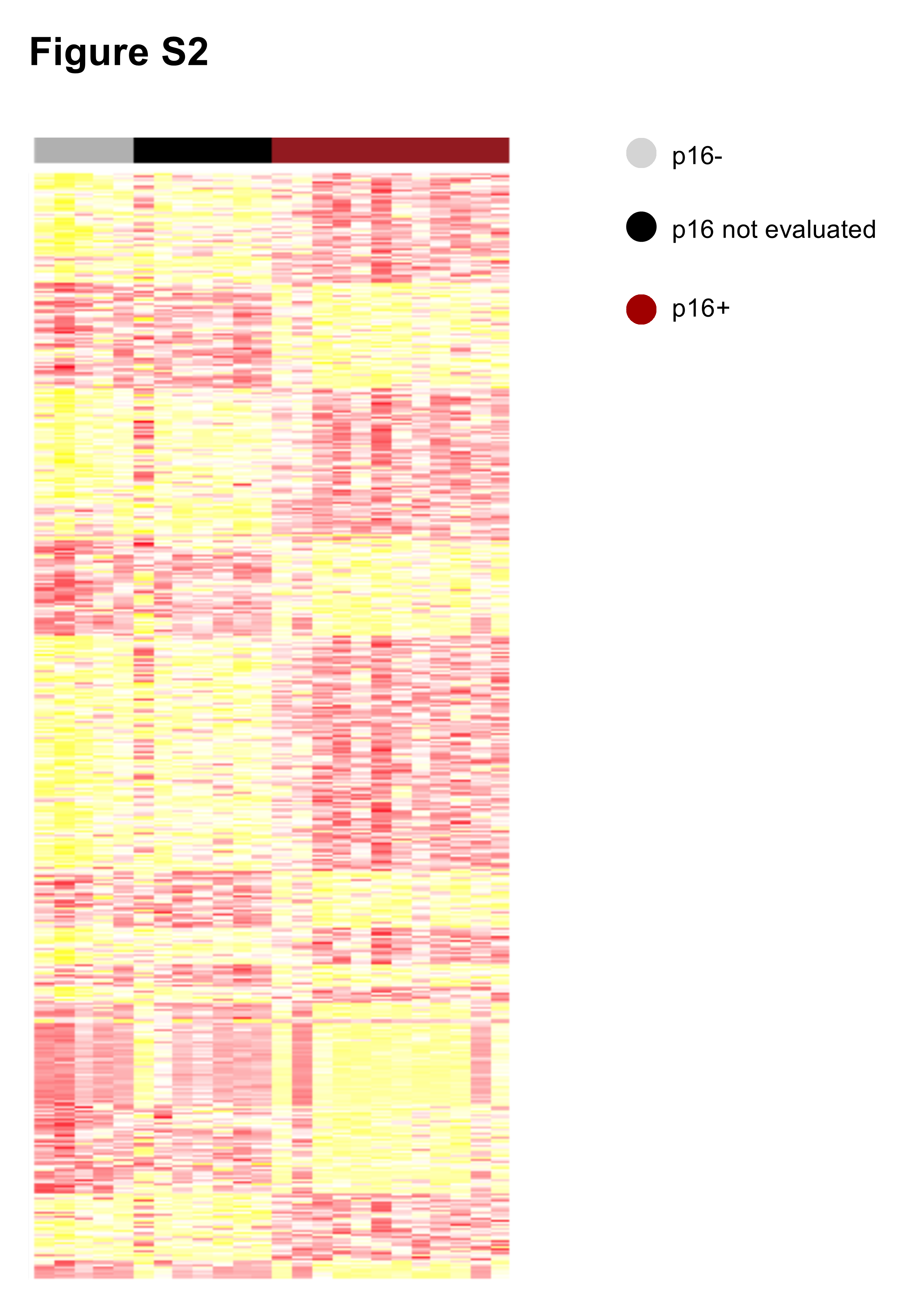

Supplement: S2 Fig — (TIF) [file pone.0238497.s008.tif]
